# Supplementary material for: Identification of photoperiod-induced specific miRNAs in the adrenal glands of Sunite sheep (Ovis aries)
Source: Front Vet Sci. 2022 Jul 22;9:888207. doi: 10.3389/fvets.2022.888207 (PMC9354845; doi:10.3389/fvets.2022.888207)
Supplement: Supplementary file 1 [file Data_Sheet_1.ZIP › Supplementary file/miRDeep2 file.zip!/miRDeep2 file/Novel_Structure/LP42A2/Novel_209.pdf]

Provisional ID	:	NC_019469.2_22275
Score total	:	1
Score for star read(s)	:	-1.3
Score for read counts	:	-1.3
Score for mfe	:	2.1
Score for randfold	:	1.6
Score for cons. seed	:	
Total read count	:	9
Mature read count	:	8
Loop read count	:	0
Star read count	:	1

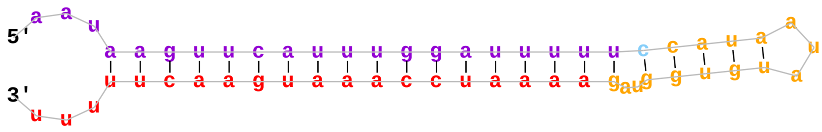

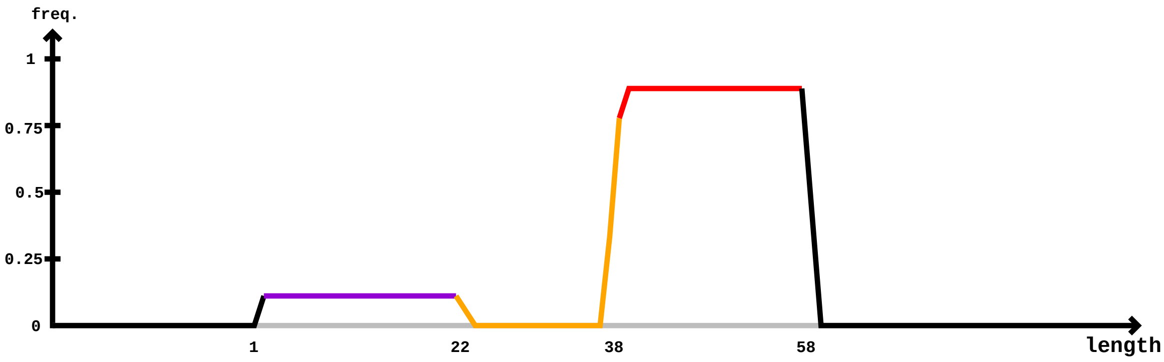

Star   Mature

```
5'- ggauugguauuggguuggccaauaaguucauuuggauuuuuccauaauaugugguagaaaauccaaaugaacuuuuuugccaacccaauagaugcaaccuauuacauauaa   -3'    obs
    ggauugguauuggguuggccaauaaguucauuuggauuuuuccauaauaugugguagaaaauccaaaugaacuuuuuugccaacccaauagaugcaaccuauuacauauaa          exp
    (.(((..((((((((((((.((.(((((((((((((((((((((((...)))))..)))))))))))))))))).)).))))))))))))))).)................    reads    mm    sample
    ....................aauaaguucauuuggauuuuu......................................................................    1        0     seq
    ........................................................gaaaauccaaaCgaacuuuuu..................................    3        1     seq
    .........................................................aaaauccaaaCgaacuuuuu..................................    3        1     seq
    .........................................................aaaaCccaaaugaacuuuuuu.................................    1        1     seq
    ..........................................................aaauccaaaCgaacuuuuuu.................................    1        1     seq
```
